# Changes in diet and physical activity following a community-wide pilot intervention to tackle childhood obesity in a deprived inner-London ward

**DOI:** 10.1186/s12889-024-18192-8

**Published:** 2024-03-14

**Authors:** Charan Bijlani, Charlotte Vrinten, Cornelia Junghans, Kiara Chang, Ellie Lewis, UmmeZeinab Mulla, Paraskevi Seferidi, Anthony A. Laverty, Eszter P. Vamos

**Affiliations:** 1 Department of Primary Care & Public Health, Public Health Policy Evaluation Unit, Imperial College London, 3rd Floor Reynolds Building, St Dunstan’s Road, London, W6 8RP UK; 2 National Institute of Health Research (NIHR) School of Public Health Research (SPHR), London, UK; 3 National Institute for Health and Care Research Applied Research Collaboration Northwest London, London, UK; 4 London Borough of Islington, London, UK

**Keywords:** Childhood obesity, Community-based participatory research, Diet, Exercise, Health improvement, Physical activity, Prevention, Obesity policy

## Abstract

**Background:**

Local authorities in England have an important role in shaping healthy local environments contributing to childhood obesity. This study examined changes in diet and physical activity in primary school children following a three-year, complex, community-based intervention in Golborne ward, the second most deprived ward in London.

**Methods:**

The Go-Golborne intervention aimed to shape the local environment across multiple settings with the engagement of a large number of local government and community stakeholders in a joint approach. Activities focused on six co-created themes to make changes to local environments and reduce sugary snacks and beverage consumption, increase fruit and vegetable intake, promote healthy snacks, increase active play and travel, and reduce screen time. We analysed changes in self-reported diet and physical activity, collected annually between 2016 and 2019, from 1,650 children aged 6–11 years through six local schools, who all received the intervention. We used multilevel, linear and logistic random-slope regression models adjusted for time on study, baseline age, gender, ethnicity, deprivation quintile, school, and baseline weight status.

**Results:**

After three years of follow-up, there were reductions in sugar-sweetened beverage consumption (adjusted beta -0·43 occasions/day, 95% CI -0·55 to -0·32), fruit and vegetable consumption (adjusted beta -0.22 portions, 95% CI -0.44 to 0.001) and car travel to and from school (adjusted OR 0·19, 95% CI 0·06 to 0·66), while screen time increased (high versus moderate/low: OR 2·30, 95% CI 1·36 to 3·90). For other behavioural outcomes, there was no statistically significant evidence of changes.

**Conclusion:**

Local authorities have substantial powers to make positive changes to the obesogenic environment but programmes remain under-evaluated. Results from the ambitious Go-Golborne intervention demonstrated mixed results in health behaviours following programme implementation. These results underline the importance of a coordinated and comprehensive policy response to support changes in wider environmental and social conditions as well as appropriate and holistic evaluations of initiatives to inform local actions on obesogenic environments.

**Supplementary Information:**

The online version contains supplementary material available at 10.1186/s12889-024-18192-8.

## Background

Globally, rates of childhood obesity are rising [1] and in England, nearly a quarter of children are overweight or obese when entering primary school, rising to 35% when children leave primary school [2]. Many children with overweight carry along this trajectory to adulthood putting them at increased risk of cardiovascular disease, cancer, type 2 diabetes, and mental ill health [1–6] Obesity is a major contributor to widening health inequalities, and childhood obesity is two-fold higher in the most deprived areas compared with the most affluent neighbourhoods in England [7–9].

Obesity is driven by obesogenic environments in the context of complex inter-relationships between numerous social, economic, physical, cultural, and policy factors [10]. To respond to the complex nature of obesity, there has been a growing interest in approaches that address health determinants at various levels (population, community, individual) [11]. In particular, addressing environmental, social, cultural, and food environments where children live, learn and play can significantly impact their dietary patterns and levels of physical activity, with important implications for their health.

Complex community-wide approaches acknowledge the significance of addressing the multifactorial drivers of obesity. These strategies aim to address obesogenic environments across various settings, presenting potential for improvement in childhood obesity rates [11, 12]. Local governments in England have substantial powers to shape local environments [13]. However, it is less clear what mechanisms could bring about changes towards health-promoting environments, and local interventions often lack evaluations with limited knowledge sharing across different areas [14].

Go-Golborne was a complex community-based intervention to prevent childhood obesity in Golborne ward, the second most deprived ward in London with a high resident population from ethnic minority backgrounds [15]. Go-Golborne served as an exploratory intervention to examine how local authority levers could be used in combination with community engagement to make structural environmental changes and how the effects of these changes may be monitored [15]. The project focused on bringing together different local government departments to support the development of the intervention. Importantly, Go-Golborne was co-designed and implemented with local authority departments, numerous local stakeholders and community members and was synchronised with existing local resources [14, 15].

This study aims to examine changes in diet and physical activity for children aged 6 to 11 years attending all six state-funded primary schools in the Golborne ward during the four years of the Go-Golborne intervention.

## Methods

### The Go-Golborne intervention

**Fig. 1:**
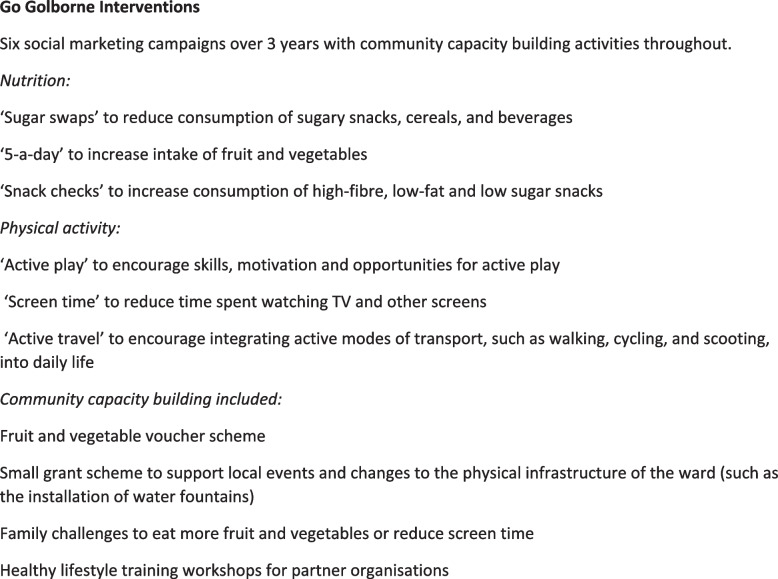
Specific Go-Golborne Interventions Implemented Between 2016–2019

The design of the Go-Golborne intervention has been described in detail elsewhere [14, 15]. Briefly, it aimed to shape the environment and behaviours across a range of settings by using local authority levers and local assets, connecting a large number of stakeholders, and synchronising on-going activities [15]. Six co-designed behaviour change targets were co-developed: three focused on dietary changes and three on physical activity changes (see Fig. 1). Go-Golborne is a prospective open cohort study, and due to the nature of funding and allocation of resources in the local authority setting, this project did not allow the use of a control group. Ethical approval for this study was granted by the University of Kent Research Ethics Approval (SRCEA 150).

### Study design

**Fig. 2:**
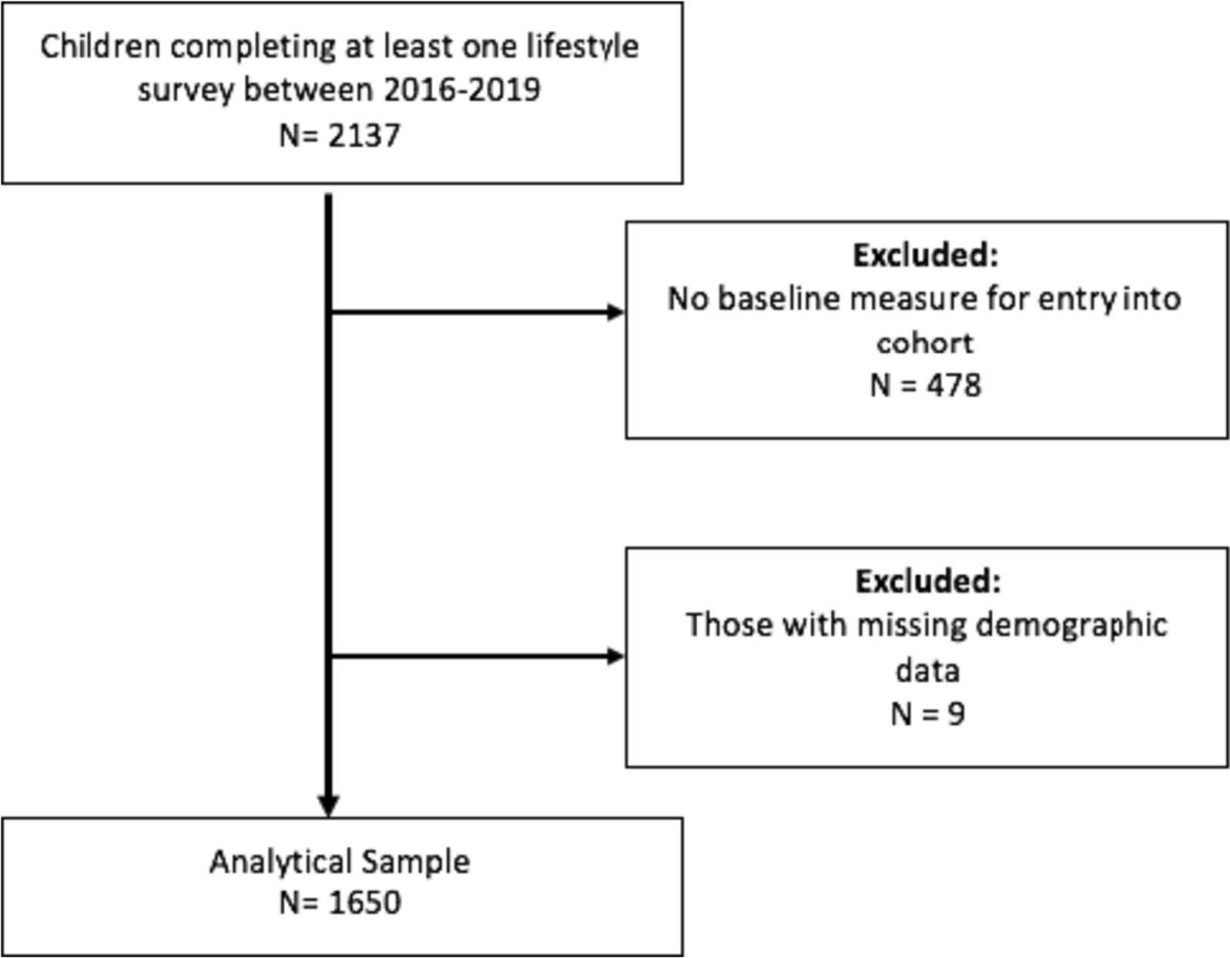
Study flow chart for study inclusion

Data collection took place in all six state-funded primary schools in the Golborne ward. All children aged 6–11 years (from Year 2 to Year 6) from these schools were invited to participate in annual data collections from 2016 to 2019. Children were given the opportunity to opt-out of survey completion before the study and decline to take part on the day the survey was conducted in schools [14, 16]. In 2016, 89.3% of children who attended six primary schools in Golborne completed a survey (*N* = 1114). We included children in the cohort who had data at baseline, including children who later became eligible for participation (i.e. entered Year 2 during the study period in 2017, 2018 or 2019). We excluded all children without a baseline measure (Fig. 2) resulting in 1,650 participants included in the analytical sample.

### Data and measures

Data were collected through annual child-completed surveys administered at school. The surveys were developed for the Go-Golborne intervention and were based on existing measures (adapted from the Day in the Life Questionnaire and Child Nutrition Questionnaire) and were tailored with use of graphics and audio files to make them appropriate for children [15]. The surveys were pilot tested before use and completed with the support of trained assistants.

Dietary outcome measures were fruit and vegetable intake, and consumption of: sugar-sweetened beverages (SSB), water, sugary snacks, and crisps. Total fruit and vegetable intake was assessed with 10 questions asking about consumption in the last 24 h during meal and snack times (Supplementary Table 1). Similarly, consumption of SSB, water, sugary snacks, and crisps were measured by summing all responses assuming each food selected represented one portion/serving. More than one portion of fruit juice was included as a SSB portion, following current UK guidelines [17] (Supplementary Table 1).

Physical activity measures included active play, school commute, and screen time. Active Play was indexed by how children responded to what they did at morning play, lunchtime play and number of after school activities (Supplementary Table 2). After examining the distribution of responses to these three items, we created a variable with three categories to index active play: high, moderate, and low (Supplementary Table 2). We further created two binary variables for the analyses: “high active play versus the rest” and “low active play versus the rest”.

“School Commute” was measured with two questions: “How do you usually travel to school in the morning/travel home after school?” Responses were combined into three categories: high [low/moderate] physical activity from the school commute. We then created two binary variables for analyses similarly to the physical activity measure (Supplementary Table 3). Screen time was assessed with three items: “On school days, how often do you usually watch TV or play on the computer before school [after school, after your evening meal]?”. From these items, a total screen time variable was created with three categories: “high [low/moderate] screen time. We again created two binary variables. (Supplementary Table 4).

#### Other study variables

The following covariates were included: age at baseline, gender (boy/girl), ethnicity, quintile of deprivation at baseline, school, and weight status at baseline. Time on study ranged from 0 (baseline) to 3 years and was modelled as a categorical variable with the baseline year as the reference group.

Ethnicity was recorded by the school nurse as part of the extended National Child Measurement Programme (NCMP), as “White”, “Black”, “Asian”, “Other” or “Missing”. Quintile of deprivation (QOD) was assigned according to the Index of Multiple Deprivation, a composite measure of neighbourhood deprivation based on household postcode [18]. We collapsed the five QOD groups into three categories with the three least deprived quintiles in one group due to small sample size. Missing observations were coded as a separate category.

Anthropometric data were collected through an extended NCMP. The national NCMP programme is a mandated opt- out service of local authorities, carried out annually that collects measurements of height and weight for children in Reception (the first year of primary school, typically aged 4–5) and Year 6 (the final year of primary school, typically aged 10–11) and is conducted by school nurses. For Go-Golborne, an extended NCMP was used that performed measurements for children of all school years during 2016–2019. Parents and carers could withdraw consent by opting out of the NCMP/extended NCMP. Weight status was assigned to each child based on gender and age-adjusted Body Mass Index (BMI) centiles. Children were classified as underweight, healthy weight, overweight, or obese according to the British 1990 Growth reference charts [19]. Due to small numbers of underweight children (1%), we combined this category with the healthy weight category.

### Analyses

Baseline characteristics are described as frequencies and proportions or means and standard deviation. Differences in characteristics between boys and girls were compared using Chi-squared test for categorical variables, t-test for normally distributed variables, and Mann–Whitney U test for skewed continuous variables, as appropriate.

We used separate 2-level linear or logistic random slope regression models to account for clustering of repeated outcome measurements within children. Multilevel linear regression models were used to examine associations between time on study and the dietary outcome measures. Multilevel logistic regression models were used to estimate in the likelihood of the physical activity outcome measures, adjusting for study covariates. Time on study was entered into the fixed and random components of the multilevel models, while all other variables were added to the fixed component. We tested the incorporation of a 3rd level with clustering of children within schools, but this did not further improve model fit. Thus, we report the 2-level models for each outcome and included school in the fixed component of the models. In subgroup analyses, we further examined whether any changes in diet or physical activity differed between groups by further including an interaction term between time on study and gender, ethnicity, weight status and deprivation in separate models.

All analyses were conducted using Stata version 16.1 (StataCorp LLC), using a two-sided alpha level of 0.05 to denote statistical significance.

## Results

### Sample characteristics

A cohort of 1,650 children were included for analysis (Table 1) [16]. Children excluded from the analyses were similar in gender but were more likely to have data missing for ethnicity, deprivation quintile and BMI compared with study participants (Supplementary Table 4).

**Table 1:** Baseline sample characteristics of the Go-Golborne cohort by gender (*N* = 1,650)

**Characteristics**	**Boys**	**Girls**	**Total**	***P*** **-Value**
	842 (51.0)	808 (49.0)	1650 (100.0)	
**Age (N, %)**				
Mean (SD)	7.8 (1.6)	7.9 (1.6)	7.9 (1.6)	0.31
**Duration of Follow Up (1–4 years)**				
Mean (SD)	1.1 (1.1)	1.0 (1.1)	1.1 (1.1)	0.24
**Ethnicity (N, %)**				
White	222 (26.4)	188 (23.3)	410 (24.9)	
Black	189 (22.5)	179 (22.2)	368 (22.3)	
Asian	41 (4.9)	45 (5.6)	86 (5.2)	0.62
Other	183 (21.7)	185 (22.9)	368 (22.3)	
Missing	207 (24.6)	211 (26.1)	418 (25.3)	
**Deprivation quintile (N, %)**				
1 (most deprived)	428 (50.8)	431 (53.3)	859 (52.1)	
2	208 (24.7)	196 (24.3)	404 (24.5)	0.35
3–5 (least deprived)	83 (9.9)	86 (10.6)	169 (10.2)	
Missing	123 (14.6)	95 (11.8)	218 (13.2)	
**School (N, %)**				
School 1	139 (16.5)	150 (18.6)	289 (17.5)	
School 2	152 (18.1)	119 (14.7)	271 (16.4)	
School 3	156 (18.5)	145 (18.0)	301 (18.2)	0.50
School 4	160 (19.0)	154 (19.1)	314 (19.0)	
School 5	102 (12.1)	106 (13.1)	208 (12.6)	
School 6	133 (15.8)	134 (16.6)	267 (16.2)	
**Weight status (N, %)**				
Underweight or healthy weight	520 (61.8)	513 (63.5)	1033 (62.6)	
Overweight	86 (10.2)	100 (12.4)	186 (11.3)	0.12
Obese	84 (10.0)	81 (10.0)	165 (10.0)	
Missing	152 (18.0)	114 (14.1)	266 (16.1)	
Fruit and vegetable intake (portions/day)*				
Mean (SD)	2.9 (2.0)	3.3 (1.9)	3.1 (1.9)	< 0.01
Sugar-sweetened beverage intake (occasions/day; range 0–5)*				
Mean (SD)	0.9 (1.1)	0.7 (1.0)	0.8 (1.1)	< 0.01
Water intake (occasions/day; range 0–5)*				
Mean (SD)	1.2 (1.3)	1.5 (1.3)	1.4 (1.3)	< 0.01
**Sugary snack intake (portions/day; range 0–8)**				
Mean (SD)	0.9 (1.4)	0.9 (1.3)	0.9 (1.3)	0.36
**Crisps intake (occasions/day, range 0–4)**				
Mean (SD)	0.5 (0.9)	0.4 (0.8)	0.5 (0.8)	0.11
**Active Play** ^**a**^				
High	136 (16.2)	110 (13.6)	246 (14.9)	
Moderate	603 (71.6)	587 (72.7)	1190 (72.0)	0.24
Low	100 (11.9)	110 (13.6)	210 (12.7)	
Missing	3 (0.4)	1 (0.1)	4 (0.2)	
**School Commute** ^**b**^				
Active travel both ways	413 (49.1)	412 (51.0)	825 (50.0)	
Multiple modes	221 (26.3)	210 (26.0)	431 (26.1)	0.70
Car travel both ways	202 (24.0)	182 (22.5)	384 (23.3)	
Missing	6 (0.7)	4 (0.5)	10 (0.6)	
**Screen Time** ^**c**^ *				
Low	363 (43.0)	394 (48.8)	757 (45.9)	
Medium	306 (36.3)	278 (34.3)	584 (35.4)	0.03
High	162 (19.2)	123 (15.2)	285 (17.3)	
Missing	11 (1.3)	13 (1.6)	24 (1.5)	

^a^Physical activity determined by morning play, lunch play and number of after school activities. High is those that run/walk at morning and lunch play and do more than 2 after school activities. Low is those that sit/stand at morning or lunch play and do 0 after school activities. Moderate is those in the middle

^b^Commute to and from school

^c^Screen time determined by those who watch TV on three occasions and the frequency. Low screen time is those on all three occasions never/not very often watch TV. High is identified as those who on all three occasions watch TV everyday/most days. Medium is those in the middle

^*^statistically significant difference between genders (*P* < 0.05)

Mean age at baseline was 7.9 years (SD 1.6 years) and 49.0% were female. A large proportion were from White ethnic backgrounds (24.9%), Black (22.3%), and other ethnic backgrounds (22.3%). Most participants were from the two most deprived quintiles (52.1% quintile 1, 24.5% quintile 2). Nearly two-thirds of children were of normal weight (62.6%), 11.3% were overweight, and 10.0% was obese at baseline. Among boys, the proportions of overweight and obese boys were both 10.0%, but there were slightly more girls with overweight (12.4%) than with obesity (10.0%). 53.3% of children were followed up for at least one year, 36.3% were followed up for at least two years, and 17.4% for all three years, with a mean follow-up time of 1.1 years (SD 1.1 years).

### Changes in dietary outcomes

Mean consumption of the five dietary outcomes are displayed in Table 1.

Adjusted multi-level regression analyses showed that the mean consumption of SSB decreased with follow-up time (Table 2). After 2 years follow up, consumption was -0.15 occasions/day (95% CI: -0.24, -0.06) lower and -0.43 occasions/day (95%CI: -0.55, -0.32) lower after 3 years of follow up when compared to baseline. Patterns of change in other dietary outcomes were mixed otherwise. Mean consumption of water was significantly higher after one year (0.18 occasions/day; 95% CI: 0.09, 0.27) but lower after 3 years of follow-up (0.34 occasions/day; 95% CI: -0.48, -0.20) compared with baseline. Mean consumption of crisps was higher (0.08 occasions/day; 95% CI: 0.01, 0.14) while mean consumption of fruit and vegetables were lower (-0.17 portions/day; 95% CI: -0.30, -0.03) compared with baseline measures at one year follow-up. However, there were no significant changes in the consumption of crips or fruit and vegetables at 2 and 3 years of follow-up. There was no evidence of changes in the mean consumption of sugary snacks throughout follow-up.

**Table 2:** Multilevel linear random slope model estimated changes in dietary outcomes, unadjusted and adjusted models*

		**Unadjusted**			**Adjusted**^a^		
	Total**	**Beta Coefficient**	**95% Confidence Interval**	***p*** **-value**	**Beta Coefficient**	**95% Confidence Interval**	***p*** **-value**
**Fruit and vegetable consumption (portions/day)Time on study**							
0 (baseline)	1634	Ref			Ref		
1 year	878	-0.20	-0.33, -0.07	< 0.01	-0.17	-0.30, -0.03	0.01
2 years	598	-0.19	-0.35, -0.04	0.02	-0.16	-0.31,	0.05
3 years	288	-0.27	-0.49, -0.05	0.02	-0.22	0.001-0.44, 0.001	0.05
**Sugar sweetened beverage consumption (occasions/day) Time on study**							
0 (baseline)	1650	Ref			Ref		
1 year	879	-0.04	-0.11, 0.04	0.33	-0.05	-0.13, 0.02	0.16
2 years	599	-0.13	-0.22, -0.05	< 0.01	-0.15	-0.24, -0.06	< 0.01
3 years	288	-0.41	-0.53, -0.29	< 0.01	-0.43	-0.55, -0.32	< 0.01
**Water consumption (occasions/day) Time on study**							
0 (baseline)	1650	Ref			Ref		
1 year	879	0.15	0.06, 0.24	< 0.01	0.18	0.09, 0.27	< 0.01
2 years	599	0.10	-0.01, 0.21	0.06	0.12	0.02, 0.23	0.02
3 years	288	-0.39	-0.54, -0.25	< 0.01	-0.34	-0.48, -0.20	< 0.01
**Crisps consumption (occasions/day) Time on study**							
0 (baseline)	1650	Ref			Ref		
1 year	879	0.08	0.02, 0.14	0.01	0.08	0.01, 0.14	0.02
2 years	599	-0.002	-0.07, 0.07	0.96	-0.01	-0.08, 0.07	0.88
3 years	288	0.002	-0.10, 0.10	0.97	0.002	-0.10, 0.10	0.97
**Sugary snack consumption (occasions/day) Time on study**							
0 (baseline)	1650	Ref			Ref		
1 year	879	0.05	-0.05, 0.15	0.33	0.05	-0.05, 0.15	0.36
2 years	599	-0.01	-0.12, 0.11	0.91	-0.01	-0.12, 0.11	0.90
3 years	288	0.04	-0.12, 0.20	0.63	0.03	-0.13, 0.20	0.68

^a^Adjusted for age, gender, ethnicity, deprivation quintile, weight status, school

### Changes in physical activity

Baseline physical activity outcomes are shown in Table 1.

In the multi-level logistic regression analyses, we found no statistically significant changes in the likelihood of active travel to and from school across the three follow-up years in the adjusted model when compared to the reference group (Table 3). However, in follow-up year 2 and year 3 the likelihood of car travel lowered by 49% (95% CI: 0.26, 0.98) and 81% (95% CI: 0.06, 0.66) as compared with the baseline. In the first two years of follow up, children were more likely to report having high active play compared with low or mixed active play levels. The adjusted odds ratios at follow up years as compared with the baseline are: 2.26 (95% CI: 1.74, 2.93), 2.27 (95% CI: 1.60, 3.23) and 1.56 (95% CI: 0.84, 2.89) with statistically significant results in both follow up year 1 and 2.

**Table 3:** Multilevel logistic random slope model estimated changes in physical activity outcomes, unadjusted and adjusted models*

		**Unadjusted**			**Adjusted**^a^		
	**Total**^b^**(%)**	**Odds Ratio**	**95% Confidence Interval**	***p*** **-value**	**Odds Ratio**	**95% Confidence Interval**	***p*** **-value**
**School commute Active Travel both ways vs. mixture or car travel (reference)Time on study**							
0 (baseline)	1640	Ref			Ref		
1 year	879	1.14	0.84, 1.53	0.41	1.188	0.88, 1.61	0.27
2 years	598	1.03	0.70, 1.53	0.87	1.092	0.73, 1.63	0.67
3 years	288	0.61	0.33, 1.11	0.10	0.623	0.34, 1.15	0.13
**School commute Car Travel both ways vs. mixture or active travel (reference) Time on study**							
0 (baseline)	1640	Ref			Ref		
1 year	879	0.74	0.49, 1.13	0.16	0.73	0.49, 1.08	0.11
2 years 3 years	598 288	0.48 0.15	0.22, 1.03 0.04, 0.67	0.06 0.01	0.51 0.19	0.26, 0.98 0.06, 0.66	0.04 0.01
**Active Play High vs. moderate or low (reference) Time on study**							
0 (baseline)	1646	Ref			Ref		< 0.01 < 0.01
1 year 2 years	879 599	2.04 2.09	1.58, 2.62 1.47, 2.96	< 0.01 < 0.01	2.26 2.27	1.74, 2.93 1.60, 3.23	
3 years	288	1.36	0.74, 2.50	0.33	1.56	0.84, 2.89	0.16
**Active Play Low vs. moderate or high (reference) Time on study**							
0 (baseline)	1646	Ref			Ref		
1 year	879	0.63	0.47, 0.84	< 0.01	0.59	0.43, 0.81	< 0.01
2 years	599	0.56	0.40, 0.80	< 0.01	0.49	0.26, 0.87	0.02
3 years	288	0.60	0.38, 0.96	0.03	0.41	0.14, 1.21	0.11
**Screen Time Low vs. moderate or high screen time (reference) Time on study**							
0 (baseline)	1626	Ref	0.78, 1.18	0.69	Ref	0.83, 1.27	0.79
1 year	874	0.96	0.11, 0.23	< 0.01	1.03	0.11, 0.24	< 0.01
2 years	598	0.16	0.07, 0.24	< 0.01	0.17	0.08, 0.27	< 0.01
3 years	288	0.13			0.14		
**Screen Time High vs. moderate and low screen time (reference) Time on study**							
0 (baseline)	1626	Ref			Ref		
1 year	874	1.23	0.94, 1.61	0.13	1.18	0.91, 1.55	0.21
2 years	598	3.39	2.39, 4.80	< 0.01	3.21	2.27, 4.54	< 0.01
3 years	288	2.48	1.45, 4.24	< 0.01	2.30	1.36, 3.90	< 0.01

^a^Adjusted for age, gender, ethnicity, deprivation quintile, weight status, school

^b^Total observations for each follow up year. Each follow up year had a different number of children to were in the cohort

Compared with baseline, the likelihood of children reporting the lowest level of screen time decreased significantly in follow up year 2 (OR: 0.17, 95% CI: 0.11, 0.24) and follow up year 3 (OR = 0.14, 95% CI: 0.08, 0.27). However, in parallel there was an increase in children in the highest levels of screen time compared with combined group of low or moderate screen time (year 2 (OR: 3.21, 95% CI: 2.27, 4.54) and year 3 (OR: 2.30, 95% CI: 1.36, 3.89).

### Subgroup analyses

Tests of interaction terms show no difference in changes of study outcomes between subgroups of gender, ethnicity, weight status, or deprivation, except for a significantly lower consumption of sugary snacks identified among the least derived (quintile 3–5) at 2-year follow-up (-0.48 occasions/day; 95% CI: -0.91, -0.05).

## Discussion

Go-Golborne was an ambitious local authority-led childhood obesity prevention programme that aimed to co-produce and implement a locally feasible intervention with community stakeholders in a deprived area of London. The annual assessment of physical and dietary targets produced mixed results. During the three years of the intervention, there were reductions in the consumption of SSB, and fruit and vegetables, and water consumption. There were reductions in car travel to and from school which were sustained over 3 years, but there was no evidence of changes in active play in the final year of the intervention, and there was evidence of increases in children with higher screen time.

The decrease in SSB consumption aligns with national trends of lower SSB consumption in children between 2016 and 2019 [20]. It is not possible to entangle how much of this reduction is due to increased actions on sugar including the announcement of the UK Soft Drink Industry Levy (SDIL) in 2016 [21], or the interventions implemented locally in Golborne ward, but previous interventions abroad, such as the EPODE [15] have also shown reductions in SSB consumption, with greater improvements in those from more deprived backgrounds [22, 23].

Our study showed small reductions in fruit and vegetable consumption with longer time on study. Although direct comparisons are not possible, nationally-representative data from the National Diet and Nutrition Survey (NDNS) for children aged 4–10 years in the UK shows that fruit and vegetable consumption did not change significantly and hovered around 200 g/day between 2008–2019 [24]. Other complex intervention evaluations such as Shape up Somerville showed no significant differences in fruit and vegetable consumption [25]. While we did not identify an increase in consumption, the qualitative process evaluation of Go-Golborne found positive changes in attitudes for fruits and vegetables [14]. These findings reinforce the need on increased access and affordability of healthier food options in deprived neighbourhoods.

A decrease with children travelling to and from school by car across the three years of the intervention was observed. This demonstrates an increase in more active modes of travel, however there was no observed increase in active travel via walking or cycling in our results which may indicate in the use of more active methods such as public transport. Nationally, rates of car travel to school have increased between 2016–2019 according to the National Travel Survey [26]. How children travel to and from school is not only determined by distance but also by the school environment and access to safe roads [27, 28], which demonstrates the importance and opportunity for cross-department collaborations such as including transport departments in the planning process [29].

High screen time levels increased in our study and other community-based studies report similar findings with children having no improvement in screen time or rates increasing [30]. Interventions which have found success with decreasing screen time suggest focusing on goal setting and positive reinforcement [31] however, this level of intervention was not the focus of Go-Golborne but rather broader behavioural changes.

Due to lack of funding and competing priorities, the impact of complex community programmes is often under-evaluated [32, 33] and where they have been, their results have been mixed [34]. The absence of evidence of detectable changes in health behaviours may include changes to the local environment not translating into behaviour change, or the lack of sustainable system-wide changes in obesogenic environments that could result in behaviour change at a population level [14, 35]. Importantly, changes in local environments, community capacity building and community networks are not captured in this analysis and identifying these changes require more holistic evaluations. It could be that some of the positive changes that have been reported because of Go-Golborne take longer to filter through into measurable behaviour change [14].

There are several strengths in both the Go-Golborne intervention and evaluation. Our study includes a large cohort of children in one of the most deprived areas in London. Data collection was conducted annually for four years, which is often unfeasible in local authority settings with changing political landscapes. The Go-Golborne intervention uniquely focused on changes to address systemic challenges within a highly deprived community. These changes aimed to reshape the local environment working with the community to create more health-promoting local spaces. This study tested how complex community-based approaches to obesity work in a real-world setting and the challenges of how communities, local authorities and researchers can work together.

Nonetheless the study has several limitations. The lack of longitudinal data from other local authorities or national surveys for study outcomes during the study period precluded making comparisons with national trends. Due to financial and ethical constraints in the local authority, incorporating a control group was not feasible for this project. Data on changes in class sizes due to children changing schools during the study period were not available, and therefore, we could not provide response rates for each study year. Furthermore, the number of children who were able to complete the three surveys resulted in a reduced sample that may not be representative of the wider population, as well as reduced the statistical power of the analyses.

Retrospective self-reporting of health behaviours is prone to recall and social desirability biases, and some studies have found only a weak agreement between self-reports of diet and physical activity in survey, food diary and accelerometer data [34]. Although the questionnaires were designed to be completed by children with assistance, its accuracy could not be ascertained. Physical activity and water intake measures were designed to be appropriate for children and focused on frequency rather than duration or volume. Absolute numbers, such as portions of fruit and vegetables consumed per day, may need to be interpreted with caution, however, although our comparison with national data collected using food diaries show similar results [24]. Additionally, important themes such as transport poverty were unable to be explored in the survey as the research tools used throughout the intervention needed to balance scientific rigour, resources, and feasibility.

## Conclusion

This ambitious and complex local authority-led childhood obesity prevention programme in a deprived inner-city area of London showed mixed results in quantitative changes in the behavioural targets of the programme. The four-year programme successfully engaged and mobilised local stakeholders in a jointly developed approach, the resulting changes may not be fully captured in this evaluation or be sufficient to shift behaviour change at a population level. Engaging and working collaboratively is crucial in the public health sector and the Go-Golborne project demonstrates how this can be done and has the potential for shifts in behaviour change. However, to measure the real impact, evaluations need to match the complexity of community-based interventions with a holistic approach to detect system-wide changes. This highlights the need for a coordinated and comprehensive local and national policy response to support changes in wider environmental and social conditions.

### Supplementary Information

**Supplementary Material 1.**

## Data Availability

The data that support the findings of this study are available from Royal Borough of Kensington and Chelsea Council (RBKC) Public Health Team, but restrictions apply to the availability of these data, which were used under license for the current study, and so are not publicly available. Data are however available from the authors upon reasonable request and with permission from the Royal Borough of Kensington and Chelsea Council (RBKC) Public Health Team. Please contact Charan Bijlani at cgill@ic.ac.uk to request the data.

## Abbreviations

BMI: Body Mass Index
NCMP: National Child Measurement Programme
NDNS: National Diet and Nutrition Survey
PA: Physical Activity
EPODE: Ensemble Prévenons l'Obésité des Enfant
QOD: Quintile of Deprivation
SSB: Sugar Sweetened Beverages
