# Supplementary material for: Changes in diet and physical activity following a community-wide pilot intervention to tackle childhood obesity in a deprived inner-London ward
Source: BMC Public Health. 2024 Mar 14;24:800. doi: 10.1186/s12889-024-18192-8 (PMC10938768; doi:10.1186/s12889-024-18192-8)
Supplement: Supplementary file 1 — Supplementary Material 1. [file 12889_2024_18192_MOESM1_ESM.docx]

**Changes in diet and physical activity following a community-wide pilot intervention to tackle childhood obesity in a deprived inner-London ward**

Charan Bijlani*^1,2^, Charlotte Vrinten*^1^, Cornelia Junghans^1,3^, Kiara Chang^1^, Ellie Lewis^4^, UmmeZeinab Mulla^1^, Paraskevi Seferidi^1^, Anthony A Laverty^1^, Eszter P Vamos^1^

*These authors contributed equally to this work

Affiliations:

^1^ Department of Primary Care & Public Health, Imperial College London, London, UK

^2^ National Institute of Health Research (NIHR) School of Public Health Research (SPHR)

^3^ National Institute for Health and Care Research Applied Research Collaboration Northwest London

^4^ London Borough of Islington

Corresponding author: Charan Bijlani, cgill@ic.ac.uk

Public Health Policy Evaluation Unit, Imperial College London

3^rd^ Floor Reynolds Building, St Dunstan’s Road, Imperial College London, London W6 8RP, United Kingdom

Abstract: 328 words (max 350)

Word count: 2937 words

Tables: 3

Figures: 2

References: 36

Supplementary files: 5

Supplementary Tables

Supplementary Table 1. Derivation of Dietary Outcome Variables

| **Question** | **Response** | **Final Variable** |
| --- | --- | --- |
| Did you have fruit with your breakfast? | “Yes” | Total Fruit/Veg |
| Did you have vegetables with your breakfast? |  |  |
| Did you have fruit with your lunch? |  |  |
| Did you have vegetables with your lunch? |  |  |
| Did you have fruit with your dinner? |  |  |
| Did you have vegetables with your dinner? |  |  |
| Did you have a snack/drink on the way to school? | “Yes” and if a fruit/vegetable was selected then included in the total |  |
| Did you have a snack/drink on the way home from school? |  |  |
| Did you have a snack/drink after school? |  |  |
| Did you have a snack/drink after your evening meal? |  |  |
| Did you have a snack/drink on the way to school? | “Yes” and if fizzy drink, milkshake, juice or other open ended response was a sugar sweetened beverage then included in the total | Total Sugar Sweetened Beverage Intake (Total SSB) |
| Did you have a snack/drink on the way home from school? |  |  |
| Did you have a snack/drink after school? |  |  |
| Did you have a snack/drink after your evening meal? |  |  |
| Did you have a drink with your evening meal? |  |  |
| Did you have a snack/drink on the way to school? | “Yes” and if water was selected then it was included in the total | Total Water Intake |
| Did you have a snack/drink on the way home from school? |  |  |
| Did you have a snack/drink after school? |  |  |
| Did you have a snack/drink after your evening meal? |  |  |
| Did you have a drink with your evening meal? |  |  |
| Did you have a snack/drink on the way to school? | “Yes” and if biscuits, sweets and other high sugar responses were listed then included in total | Total Sugary Snack Intake |
| Did you have a snack/drink on the way home from school? |  |  |
| Did you have a snack/drink after school? |  |  |
| Did you have a snack/drink after your evening meal? |  |  |
| Did you have a snack/drink on the way to school? | “Yes” and if crisps were selected then included in total | Total Crisp Intake |
| Did you have a snack/drink on the way home from school? |  |  |
| Did you have a snack/drink after school? |  |  |
| Did you have a snack/drink after your evening meal? |  |  |

Supplementary Table 2. Derivation of Active Play Variable

|  | **Do at Lunch Play** |  |  |  |  |
| --- | --- | --- | --- | --- | --- |
| **Do at Morning Play** | Sit | Stand | Walk | Run | **Number of After School Activities** |
| Sit | Low | Low | Moderate | Moderate | 0 |
| Stand | Low | Low | Moderate | Moderate |  |
| Walk | Moderate | Moderate | Moderate | Moderate | 1 |
| Run | Moderate | Moderate | Moderate | High | 2 |

Supplementary Table 3. Derivation of School Commute Variable

|  | **“Commute from School”** |  |  |  |
| --- | --- | --- | --- | --- |
| **“Commute to School”** | Walk/Run | Cycle/Scoot | Public Transport | Car |
| Walk/Run | Active travel | Active travel | Multiple modes | Multiple modes |
| Cycle/Scoot | Active travel | Active Travel | Multiple modes | Multiple modes |
| Public transport | Multiple modes | Multiple modes | Multiple modes | Multiple modes |
| Car | Multiple modes | Multiple modes | Multiple modes | Car use |

Supplementary Table 4. Derivation of Screen Time Variable

|  | **TV Before Dinner** |  |  |  |  |
| --- | --- | --- | --- | --- | --- |
| **TV Before School** | Everyday | Most days | Not very often | Never | **TV After Dinner** |
| Everyday | High | Moderate | Moderate | Moderate | Everyday |
| Most days | Moderate | Moderate | Moderate | Moderate | Most days |
| Not very often | Moderate | Moderate | Low | Low | Not very often |
| Never | Moderate | Moderate | Low | Low | Never |

Supplementary Table 5. Characteristics for Excluded Participants (N=487)

|  | Participants Excluded | Participants Included |  |
| --- | --- | --- | --- |
| Characteristics | N (%) | N (%) | P-value |
| Gender  Male  Female  Missing | 257 (52.8)  229 (47.0)  1 (0.2) | 842 (51.0)  808 (49.0)  0 (0.0) | 0.473^1^ |
| Ethnicity  White  Black  Asian  Other  Missing | 76 (15.6)  75 (15.4)  31 (6.4)  48 (9.9)  257 (52.8) | 410 (24.9)  368 (22.3)  86 (5.2)  368 (22.3)  418 (25.3) | <0.001^1^ |
| Deprivation quintile  1 (most deprived)  2  3-5 (least deprived)  Missing | 231 (47.4)  94 (19.3)  42 (8.6)  120 (24.6) | 859 (52.1)  404 (24.5)  169 (10.2)  218 (13.2) | <0.001^1^ |
| School  School 1  School 2  School 3  School 4  School 5  School 6  Missing | 89 (18.3)  86 (17.7)  58 (11.9)  75 (15.4)  31 (6.4)  139 (28.5)  9 (1.9) | 289 (17.5)  271 (16.4)  301 (18.2)  314 (19.0)  208 (12.6)  267 (16.2)  0 | <0.001^1^ |
| Weight status  Under/healthy weight  Overweight  Obese  Missing | 245 (50.3)  37 (7.6)  74 (15.2)  131 (26.9) | 1033 (62.6)  186 (11.3)  165 (10.0)  266 (16.1) | <0.001^1^ |

^1^p-value determined from chi-square test statistic (p-value)
